# Tyrol Petitions for a Veterinary School

**Published:** 1896-09

**Authors:** 


TYROL PETITIONS FOR A VETERINARY SCHOOL. At the session of the Tyrol Landtag, February 12, 1896, among other things the following petition was unanimously adopted without debate:

The Imperial Government is most urgently requested to provide for the establishment of a veterinary school for the Alp lands at the expense of the State. This school has been already for years a pressing need of the cattle-breeders, and has been petitioned for again and again in vain. The people of Tyrol wish a veterinary institute in their own land, and do not mean to be satisfied with a mere extension of the Vienna school.—*Thierärzt. Centralblatt*, March 1, 1896.

